# Hydrolyzed Protein Formula for Allergy Prevention in Preterm Infants: Follow-Up Analysis of a Randomized, Triple-Blind, Placebo-Controlled Study

**DOI:** 10.3389/fped.2020.00422

**Published:** 2020-07-30

**Authors:** Antonio Di Mauro, Maria Elisabetta Baldassarre, Giulia Brindisi, Anna Maria Zicari, Martina Tarantini, Nicla Laera, Manuela Capozza, Raffaella Panza, Silvia Salvatore, Licia Pensabene, Margherita Fanelli, Nicola Laforgia

**Affiliations:** ^1^Section of Neonatology and Neonatal Intensive Care Unit, Department of Biomedical Science and Human Oncology, “Aldo Moro” University of Bari, Bari, Italy; ^2^Pediatrics Department, Umberto I Hospital, Sapienza University, Rome, Italy; ^3^Department of Pediatric, Ospedale “F. Del Ponte”, University of Insubria, Varese, Italy; ^4^Pediatric Unit, Department of Medical and Surgical Sciences, University “Magna Graecia” of Catanzaro, Catanzaro, Italy; ^5^Department of Interdisciplinary Medicine, “Aldo Moro” University of Bari, Bari, Italy

**Keywords:** preterm/full term infants, infant formula, hydrolyzed protein formula, hypersensitivity, allergy

## Abstract

**Background:** Allergic diseases are a major public health burden worldwide. Evidence suggests that early nutrition might play a key role in the future development of allergies and the use of hydrolyzed protein formulas have been proposed to prevent allergic disease, mainly in term infants with risk factors.

**Aim:** To evaluate the preventive effect of a hydrolyzed protein formula vs. an intact protein formula on allergy development in preterm infants with or without risk factors.

**Methods:** We performed a 3-year follow-up study of a previous triple-blind, placebo-controlled randomized trial. Evidence of atopic dermatitis, asthma and IgE-mediated food allergies were evaluated according to a validated parental questionnaire (Comprehensive Early Childhood Allergy Questionnaire). Food sensitization was also investigated by skin prick test at 3 years of chronological age.

**Results:** Of the 30 subjects in the intact protein formula group and 30 in the extensively hydrolyzed formula group, respectively 18 and 16 completed the 3-year follow-up and entered the final analysis. No group differences in the incidence of atopic dermatitis, asthma, IgE-mediated food allergies, and food sensitization were found.

**Conclusion:** Despite the small number of cases, extensively hydrolyzed protein formula seems to be ineffective in allergic diseases prevention in preterm neonates. Further adequately powered, randomized controlled trials evaluating hydrolyzed protein formula administration to prevent allergic diseases in preterm neonates are needed.

## Introduction

Allergic rhinitis (AR), asthma, eczema, and food allergy are some of the most common pediatric chronic conditions worldwide and have a major impact on children health and quality of life (1).

Allergic diseases are genetically determined but also influenced by several factors such as environmental pollution, smoke, aeroallergens, and early feeding pattern (2). To date, a tailored approach seems to be the best strategy to hamper the so-called “atopic march” (3). In this perspective, standard operative procedures to prevent allergy have become a priority in managing public health and infant feeding is considered the most important modifiable factor that can be targeted (4).

World Health Organization (WHO) states that breast milk is the best source of nutrients for both term and preterm infants, and there is some evidence of its role in decreasing the risk of allergy development (5, 6). Unfortunately human milk is not always available and the challenge for many pediatric societies remains to draw up standardized and definitive guidelines to recommend the most effective infant formula in allergy prevention (7).

More recently, hydrolyzed formulas (HF) have been proposed for prevention of allergy and many studies suggested the use of these formulas in formula-fed infants with a family history of allergic diseases (8).

The main differences between each HF are the degree and method of hydrolysis, with consequent different immunological, clinical, and nutritional effects: extensively hydrolyzed formulas (eHF) contain mostly peptides ≤3 kD, while partially hydrolyzed formulas (pHF) ≤5 kD (9).

Despite preterm infants could be at higher risk for food allergy because of their increased intestinal permeability and their possible higher food antigen uptake, they do not show higher incidence of allergic diseases when compared to term infants (10, 11).

At the moment, human milk represents the best source of nutrients for preterm infants for its bioactive effect (12). On the contrary, there is limited evidence regarding nutritional preventive action against the future development of allergies in this vulnerable population (13). This paper describes the follow-up results of a previous published triple-blind, controlled, clinical trial, in preterm neonates fed with either intact protein formula or extensively hydrolyzed formula (14, 15).

## Methods

Study design, inclusion and exclusion criteria, randomization and study group allocation, and feeding protocol are thoroughly described in the previous articles (14, 15).

In brief, all mothers were encouraged to exclusively breastfeed and to have an unrestricted diet during lactation. At birth all eligible preterm neonates, regardless family history of allergy, were randomized to receive one of two different blinded formulas: either preterm intact protein formula [IPF: marketed Enfamil® Premature, Mead Johnson Nutrition (MJN), Evansville, IN, USA] or infant extensively hydrolyzed protein formula (eHF: marketed Pregestimil®, MJN, Evansville, IN, USA). When breastfeeding was not sufficient, one of the two formulas, according to randomization, was given for 2 weeks. The research protocol was approved by the ethical committee of “Azienda Ospedaliero—Universitaria Consorziale Policlinico” (number 4122—date 17/2/2016). Parents or legally authorized representatives provided written informed consent prior to enrolment.

To all participants, complementary feeding was recommended after the age of 4 months, without restriction of possible allergenic foods and with intact protein milk formulas in case of insufficient breast milk. To compare the allergy-preventive effect of eHF vs. IPF, all infants were followed-up 6-monthly until 3 years of chronological age, for evidence of atopic dermatitis, asthma, and IgE-mediated food allergies according to a validated parental questionnaire (Comprehensive Early Childhood Allergy Questionnaire) (16). Food sensitization, based on positive skin prick tests at 3 years of chronological age, was also investigated.

### Statistical Analysis

Participant characteristics at enrolment were compared by Student *t*-test (gestational age and birth weight) or chi-square test (gender, birth type, cesarean section). Outcomes such as evidence of atopic dermatitis, asthma, and IgE-mediated food allergies were analyzed by chi-square test. All participants who met study entrance criteria and completed the 3 years follow-up period were evaluated. A subset analysis was carried out to assess participants at high-risk for allergy. High-risk infants were defined as having at least one parent or a single first-degree relative with a history of allergic disease. All tests were conducted at α = 0.05. All analyses were conducted using IBM® SPSS® Statistics 23.

## Results

**Figure 1:**
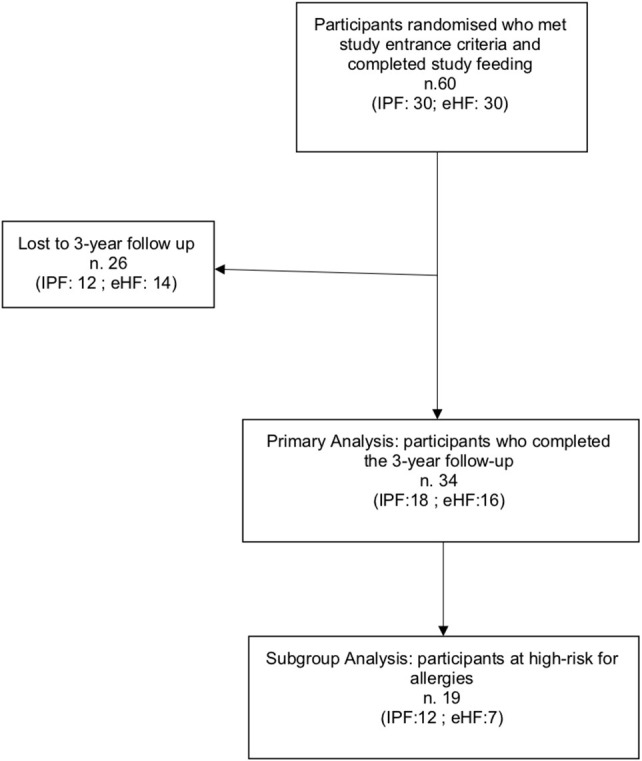
Flow chart of the study recruitment process.

A total of 34/60 (56.6%) participants completed the 3-year follow-up study (IPF: 18; eHF: 16) and were included in the primary analysis. Dropouts occurred in 26 children due to protocol violation (3 patients) and voluntary withdrawal by parents during the follow-up period (23 patient) (Figure 1).

A subset analysis among infants at high risk for allergy included 19 participants: IPF *n* = 12 (66.7%); eHF *n* = 7 (43.8%); *p* = 0.17. Study flow chart is shown in Figure 1.

Infant characteristics at 3 years of chronological age were similar between groups (Table 1).

**Table 1:** Infant characteristics.

	**IPF**	**eHF**	***p***
**Gender**, ***n*** **(%)**			0.774
Male	11 (61%)	9 (56%)	
Female	7 (39%)	7 (44%)	
**Birth type**, ***n*** **(%)**			0.311
Singleton	7 (39%)	9 (56%)	
Twin	11 (61%)	7 (44%)	
Gestational age, weeks, mean (*SD*)	30 (1,7)	29,8 (2)	0.860
Birth weight g, mean (*SD*)	1303 (254)	1334 (244)	0.723
High risk for allergy, *n* (%)	12 (67%)	7 (44%)	0.179
**Breastfeeding duration**, ***n*** **(%)**			0.725
Exclusively formula	2 (11%)	2 (14%)	
Breastfeeding <4 months	12 (67%)	8 (53%)	
Breastfeeding >4 months	4 (22%)	5 (33%)	

No difference in incidence of atopic dermatitis, asthma, IgE-mediated food allergies and food sensitization (positive skin prick test) were detected (primary or subset analysis) between groups (Table 2).

**Table 2:** Study outcomes.

**Outcomes**	**Primary analysis**		***p*-value Fisher Exact Test**	**Subset analysis**		***p*-value Fisher Exact Test**
	**IPF n. 18**	**eHF n. 16**		**IPF n.12**	**eHF n. 7**	
Atopic dermatitis, *n* (%)	3 (17%)	3 (18%)	0.61	3 (25%)	0 (0%)	0.22
Asthma, *n* (%)	2 (11%)	2 (12%)	0.65	2 (17%)	1 (14%)	0.70
IgE-mediated food, *n* (%)	1 (6%)	1 (6%)	0.72	1 (8%)	1 (14%)	0.61
Food sensitization (positive skin prick test), *n* (%)	2 (11%)	2 (12%)	0.65	2 (17%)	2 (29%)	0.47

## Discussion

The results of our study indicated that eHFs did not provide any preventive effects of allergy in preterm infants. Despite the low number of patients and the inadequate sample size, our findings are in keeping with the most recent meta-analysis regarding HF effect on allergy prevention in term neonates. In 2015, Boyle et al. found no consistent evidence to support the use of HF formula for reducing risk of allergic diseases (17). Similarly, Osborn et al. in a 2018 Cochrane found no difference in allergic diseases such as asthma, eczema, rhinitis, and food allergy in infants fed with a HF compared to IPF (18).

Only another randomized, double-blind study was conducted in high-risk preterm infants by Szajewska et al. (19). They failed to demonstrate a decrease in the incidence of allergic diseases, yet highlighted only a preventive effect of eHF on atopic dermatitis (AD) during a short follow-up of 12 months.

Conversely, several studies have been conducted in the last decades to investigate the preventive role of HFs on allergy development in term infants (Table 3).

**Table 3:** Summary of RCTs assessing the role of different HF on allergic diseases.

**First author and year**	**Characteristics of population**	**Primary outcome**	**Secondary outcomes, if any**	**Types of the milk formula used**	**Techniques used**	**Main results**
Zeiger et al. (20)	288 high-risk infants	To evaluate the effect of: Maternal or infant food allergen avoidance on AM Sensitization and serum IgE levels at 3 and 4 years of age lgG BLG and IgG OVA responses from birth to 2 years of age	To detect the interaction between genetics and environment on atopy development	eHF-C (Nutramigen)	SPTs Total IgE sIgE Nasal eosinophil determination Specific IgG BLG and IgG OVA DBPCFC	Maternal or infant food allergen avoidance decreased the cumulative prevalence of both food allergy and food sensitization (SPTs), conversely The period prevalence of food allergy or food SPT at 3 and 4 years of age Did not undergo any changes Cow milk avoidance associated with the use of breast milk and/or casein hydrolysate formula from birth to 1 year of age, reduced significantly IgG BLG response from 4 months to 2 years of age Egg avoidance has been slightly more effective in reducing IgG response to OVA, compared to standard feeding practices, which was evident at 2 years of age Influence of genetic and environmental factors toward serum IgE levels
Mallet et al. (21)	177 high-risk infants	To assess the allergy preventive effect of eHF-C in high-risk infant (evaluated at 4,12, 24, and 48 months of age)		eHF-C CMF	Total IgE sIgE	A preventive effect on the prevalence of eczema but not of asthma in eHF-C group
Halken et al. (22)	158 high-risk infants	To examine whether eHF-W (Profylac) is as protective as eHF-C (Nutramigen) considering the development of CMA and AM until 18 months of age		eHF-C (Nutramigen) eHF-W (Profylac)	Oral challenge	The use of eHF-W and eHF-C during the first 6 months of life reduces the incidence of CMA until 18 months of life
Vandenplas et al. (23)	58 high-risk infants	To evaluate the long term effect of pHF-W on the prophylaxis of AM		pHF-W CMF	-SPTs -Total IgE -sIgE	Reduced prevalence of eczema and incidence of diarrhea in the first 6 months of life in the group fed with pHF-W
Odelram et al. (24)	91 high-risk infants	To compare eHF-W with CMF in preventing the development of atopy	To compare growth with the use of eHF-W (Profylac) and CMF	eHF-W (Profylac) CMF	-SPTs -total IgE -sIgE	Despite the lack of strong evidence in the prevention of AM and sensitization, eHF-W seems to be a valid aid in the first 6 months of age No differences between eHF-W (Profylac) and CMF considering weight gain and growth
Oldaeus et al. (25)	155 high-risk infants	To compare AD incidence and allergic sensitization during the first 18 months of life in high-risk infants, fed with eHF, pHF, or CMF from weaning up to 9 months of age		pHF-W eHF-C (Nutramigen) CMF	-SPTs -sIgE	Allergy preventive effect of eHF but not of pHF during the first 18 months of life
Halken et al. (26)	595 high-risk infants	To compare the allergy preventive effect of pHF–W with two eHFs: eHF-C (Nutramigen), and eHF-W (Profylac)	To confirm the absence of differences in the allergy preventive effect of the two eHFs, eHF-C (Nutramigen), and eHF-W (Profylac)	pHF-W eHF-C (Nutramigen) eHF-W (Profylac)	-SPTs -sIgE	Less effectiveness of pHF-W than eHFs in the prevention of CMA No difference regarding the development of AM between the two eHFs, eHF-C (Nutramigen) and eHF-W (Profylac)
von Berg et al. (27)	2,252 high-risk TERM infants (hereditary risk of atopy)	To evaluate the preventive role of HFs compared with standard CMF in the development of AM at 1 year of age (AD, FA-GIT, and urticaria)		eHF-C eHF-W pHF-W CMF	SCORAD method SPT sIgE Oral challenge	Protective effect of HFs (eHF-C and pHF-W) against AD and AM in the first year of life
Szajewska et al. (19)	122 high-risk PRETERM infants (hereditary risk of atopy)	To evaluate whether the use of HFs may prevent the development of AM (AD, GI symptoms, or wheezing) within 2–5 and 12 months of age	To measure the percentages of preterm neonates who interrupted the intervention due to formula non-acceptance or were lost for any reason at the follow-up	e-HF pHF Standard preterm formula	Total IgE sIgE	Preventive effect of eHF on AD at 12 months Increased risk for preterm infants who had been fed with eHF and then interrupted for any reason and particularly for non-acceptance of the formula
Lowe et al. (28)	620 high-risk infants (hereditary risk of atopy)	To determine whether pHF-W or soy formula modify the risk of the development of AM (eczema and food reaction) up to 2 years of age	To assess the individual incidence of eczema and food reaction in the first 2 years of life and SPT reactivity at 6, 12, and 24 months To detect the prevalence of AR, eczema and asthma at 6 and 7 years of age	pHF-W Soy formula CMF	SPTs to a *milk, egg, peanut, dust mite, rye grass, and cat dander* performed at 6, 12, and 24 months	No evidence that pHW-F or soy formula reduced the risk of AM in the first 2 years of life No evidence of reduced risk of SPT reactivity No evidence of reduced risk for AR, eczema, and asthma at 6 and 7 years of age
von Berg et al. (29)	1,451 (from the original total of 2,252 high-risk TERM infants)	To evaluate the effect of the early use of HFs on the development of AM (AD, AR, and asthma) at school age		eHF-C eHF-W pHF-W CMF	ISAAC questionnaire SCORAD method sIgE	Significant reduction of only AD at 10 years of age in children who had been fed with eHF-C or pHF-W in the early stage of their life, without a preventive effect on asthma or AR
von Berg et al. (30)	1,377 (from the original total of 2,252 high-risk TERM infants)	To assess the relationship between early use of HFs and the development of AR, asthma, and eczema up to adolescence	To detect allergic sensitization through sIgE and respiratory function (spirometry)	eHF-C eHF-W pHF-W CMF	ISAAC questionnaire SCORAD method sIgE Spirometry	Preventive effect of eHF-C (mainly) and pHF-W on eczema, AR, and asthma up to adolescence

*CMF, cow's milk formula; CMA, cow's milk allergy; eHF-W, extensively hydrolyzed whey formula; eHF-C, extensively hydrolyzed casein formula; pHF-W, partially hydrolyzed whey formula; AM, allergic manifestations; AD, atopic dermatitis; AR, allergic rhinitis; FA-GIT, food allergy with manifestation in the gastrointestinal tract; GI, gastrointestinal; ISAAC, International Study on Asthma and Allergy in Childhood; sIgE, specific IgE; SPTs, skin prick tests; OFC, Oral Food Challenge; CMF, cow's milk formula; DBPCFC, double-blind placebo-controlled food challenges*.

A preventive effect of eHF both on food allergy and sensitization was first outlined in 1992, in the RCT conducted by Zeiger et al. on 288 high-risk infants (20).

This result, despite the lack of strong evidence, was later confirmed by Odelram et al. (24) and Oldaeus et al. (25). Mallet et al. in their study on 177 high-risk infants found a preventive effect on eczema linked to the early use of eHF, without any effect on asthma (21).

Similar results on eczema were found by Vandenplas et al., in a RCT on 58 high-risk infants (23).

Another RCT on 158 high-risk infants was conducted by Halken et al., who found a reduction of CMA in the first 6 months of life associated to the early use of eHFs (whey or casein) (22).

Subsequently these results were confirmed by the same authors in a larger study on 595 high-risk infants randomized to receive eHF-W, eHF-C, or pHF (26).

The German Infant Nutritional Intervention (GINI) study is to date the largest, spontaneous, quasi-randomized trial in which 2,252 children (with a family history of allergic diseases) were randomized to receive extensively hydrolyzed casein formula (eHF-C), extensively hydrolyzed whey formula (eHF-W), partially hydrolyzed whey formula (pHF-W), or standard cow's milk formula, in order to evaluate the possible preventive role of these formulas in the development of allergic diseases during a long term follow-up (27, 29–32). They found a protective effect of eHF-C and pHF-W against AD at 1 year follow-up and a significant reduction of AD at 7–10 years of age in children. However, no preventive effect against asthma or AR was shown. These results were confirmed in the 15-year follow-up study, where the authors reported also fewer diagnoses of AR and asthma in those children fed with hydrolysates (mainly eHF-C) in their early stages of life, as if the preventive effects of HF on these two pathologies had occurred later in time. Throughout the follow-up period eHF-W did not show any preventive effects toward allergic diseases and none of the HF had influence on IgE sensitization.

Differently from GINI study, the Melbourne Atopic Cohort Study, conducted on 620 high-risk infants randomized to pHF-W, soy formula or cow's milk formula, showed neither significant difference in allergic outcomes (eczema and food reactions) in the first 2 years of life nor evidence of reduced risk of SPT reactivity and of lower risk for AR, eczema and asthma up to 6–7 years of age (28).

Furthermore, in an observational population-based study, Goldsmith et al. examined the possible association between the development of food allergy at 1 year of age and either duration of exclusive breastfeeding or use of pHF (33). They found that the incidence of food allergy was not reduced by either the duration of exclusive breastfeeding or by the use of pHF, suggesting that allergen avoidance may not be helpful in allergy prevention.

Pooling data on HF in meta-analyses is problematic due to the heterogeneity of HF products and the different sources of proteins, hence different HF should not be considered equivalent. For this reason, Szajewska et al. conducted a meta-analysis taking into consideration exclusively studies using a unique 100%-whey pHF. They found a preventive effect against all allergic diseases and eczema (34). Based on all data from the literature, use of pHF-W formula has been considered safe in healthy term neonates (35, 36).

At present, the conclusions of guidelines and meta-analysis on the role of HF for prevention of allergic diseases differ in term of recommendations, outcomes, and target population. According to some pediatric Societies the use of pHF is still indicated in infants at high risk of allergy, when mother's milk is not available or is insufficient (37, 38). Differently, other Societies, based on emerging evidence, changed their previous recommendations and no longer proposed HF for prevention of allergic diseases (39–41).

The aim of this paper was to analyse the effect of HF on allergy prevention in preterm infants using follow-up data of a previous randomized, triple-blind, placebo-controlled study. To our knowledge, this is the first long-term follow-up paper concerning allergy prevention with HF in preterm infants. We acknowledge a possible limitation of our literature search due to the search restriction for preterm infants. In fact, using Medical Subject Headings-Terms for preterm infants, we could have missed studies in which subgroup analysis of preterm infants have been carried out, but not mentioned in the title or abstract. However, in our view, publication bias could be negligible. The main limitations of the present study can be considered the underpowered number of preterm enrolled, the short period of eHF administration, the drop-out rate and the lack of quantitative diagnostic methods used to diagnose allergy other than a validate questionnaire and SPT.

To sum up, further well-designed large studies in preterm infants should be conducted to address the preventive effects of HF for allergic diseases and the nutritional non-inferiority of preterm HF compared to modern preterm IPF in these vulnerable population. Moreover, data on long term safety of HF in preterm infants and cost/benefit ratio analysis are needed.

Evidences of hypersensitivity have been described also as predisposing factor in patients with functional gastrointestinal diseases (42), whose high prevalence have been recently found in preterm newborns (43). Despite self-limited diseases, a preventive intervention for FGIDs, especially for high-risk population, might have important clinical, and socioeconomical effects (44, 45). HFs have been investigated as dietary modification for management of these conditions with inconsistent evidences, despite some authors suggested a decreased incidence in infants fed with pHF (46, 47).

Finally, bio-effective agents such as probiotic have been recently added to HF to enhance their putative role in allergic disease prevention (48) and should be evaluated since preterm infants could be considered a strategy population for their well-known dysbiosis-related conditions (49, 50).

## Conclusion

To date many rigorous systematic reviews and meta-analyses evaluating term infants concluded that evidence is not robust to support the use of HF to prevent atopic diseases. Our data did not show any allergy preventive effect of eHF in a small cohort of preterm infants and highlighted the need of further large studies to better clarify the possible role of HF for allergy disease prevention in this population.

## Data Availability Statement

The raw data supporting the conclusions of this article will be made available by the authors, without undue reservation.

## Ethics Statement

The studies involving human participants were reviewed and approved by Policlinico di Bari Ethics Committee. Written informed consent to participate in this study was provided by the participants' legal guardian/next of kin.

## Author Contributions

MB conceptualized and designed the study. MT and NLae assessed study participants and collected study data. MF performed statistical analyses. AD interpreted data and drafted the initial manuscript. GB and AZ performed literature search. MC, LP, SS, and RP revised the manuscript. NLaf coordinated and supervised all activities. All authors contributed to the intellectual content, reviewed and revised the manuscript, and approved the final version. All authors contributed to the article and approved the submitted version.

## Conflict of Interest

The authors declare that this study received funding from Mead Johnson Nutrition in order to independently enroll infants and coordinate the original study. The funder was not involved in the study design, collection, analysis, interpretation of data, the writing of this article, or the decision to submit it for publication.

## Abbreviations

AD: atopic dermatitis
AR: Allergic rhinitis
CM: Cow's milk
CMA: Cow's milk allergy
eHF-C: extensively hydrolyzed casein formula
eHF: extensively hydrolyzed formula
FGIDs: functional gastrointestinal diseases
HF: hydrolyzed formula
HM: human milk
IPF: intact protein formula
pHF-W: partially hydrolyzed whey formula
pHF: partially hydrolyzed formula
RCT: randomized controlled trials
SPT: skin prick test
VLBW: very low birth weight
WHO: World Health Organization.
